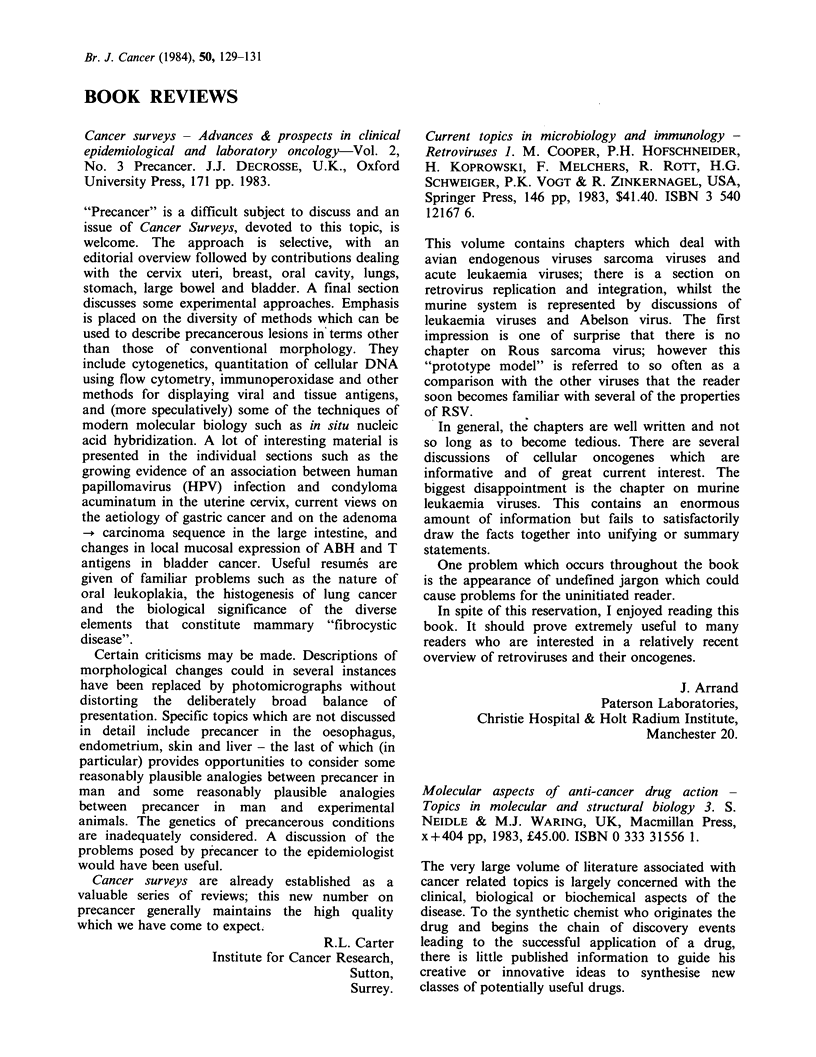# Cancer surveys - Advances & prospects in clinical epidemiological and laboratory oncology

**Published:** 1984-07

**Authors:** R.L. Carter


*Br. J. Cancer* (1984), **50**, 129–131

### BOOK REVIEWS

*Cancer surveys – Advances & prospects in clinical epidemiological and laboratory oncology*—Vol. 2, No. 3 Precancer. J.J. DECROSSE, U.K., Oxford University Press, 171 pp. 1983.

"Precancer" is a difficult subject to discuss and an issue of *Cancer Surveys*, devoted to this topic, is welcome. The approach is selective, with an editorial overview followed by contributions dealing with the cervix uteri, breast, oral cavity, lungs, stomach, large bowel and bladder. A final section discusses some experimental approaches. Emphasis is placed on the diversity of methods which can be used to describe precancerous lesions in terms other than those of conventional morphology. They include cytogenetics, quantitation of cellular DNA using flow cytometry, immunoperoxidase and other methods for displaying viral and tissue antigens, and (more speculatively) some of the techniques of modern molecular biology such as *in situ* nucleic acid hybridization. A lot of interesting material is presented in the individual sections such as the growing evidence of an association between human papillomavirus (HPV) infection and condyloma acuminatum in the uterine cervix, current views on the aetiology of gastric cancer and on the adenoma → carcinoma sequence in the large intestine, and changes in local mucosal expression of ABH and T antigens in bladder cancer. Useful resumės are given of familiar problems such as the nature of oral leukoplakia, the histogenesis of lung cancer and the biological significance of the diverse elements that constitute mammary "fibrocystic disease".

Certain criticisms may be made. Descriptions of morphological changes could in several instances have been replaced by photomicrographs without distorting the deliberately broad balance of presentation. Specific topics which are not discussed in detail include precancer in the oesophagus, endometrium, skin and liver – the last of which (in particular) provides opportunities to consider some reasonably plausible analogies between precancer in man and some reasonably plausible analogies between precancer in man and experimental animals. The genetics of precancerous conditions are inadequately considered. A discussion of the problems posed by precancer to the epidemiologist would have been useful.

*Cancer surveys* are already established as a valuable series of reviews; this new number on precancer generally maintains the high quality which we have come to expect.

R.L. Carter
Institute for Cancer Research,
Sutton,
Surrey.